# Audit and feedback in mental healthcare: staff experiences

**DOI:** 10.1108/IJHCQA-08-2017-0142

**Published:** 2018-08-13

**Authors:** Monica Stolt Pedersen, Anne Landheim, Merete Møller, Lars Lien

**Affiliations:** 1 Norwegian National Advisory Unit on Concurrent Substance Abuse and Mental Health Disorders, Innlandet Hospital Trust, Ottestad, Norway; 2 Faculty of Medicine, University of Oslo, Oslo, Norway; 3 Innland University of Applied Sciences, Campus Elverum, Elverum, Norway; 4 Østfold Hospital Trust, Norway

**Keywords:** Evidence-based practice, Quality improvement, Audit, Clinical guidelines, Self-assessment

## Abstract

**Purpose:**

Audit and feedback (A&F) often underlie implementation projects, described as a circular process; i.e. an A&F cycle. They are widely used, but effect varies with no apparent explanation. We need to understand how A&F work in real-life situations. The purpose of this paper, therefore, is to describe and explore mental healthcare full A&F cycle experiences.

**Design/methodology/approach:**

This is a naturalistic qualitative study that uses four focus groups and qualitative content analysis.

**Findings:**

Staff accepted the initial A&F stages, perceiving it to enhance awareness and reassure them about good practice. They were willing to participate in the full cycle and implement changes, but experienced poor follow-up and prioritization, not giving them a chance to own to the process. An important finding is the need for an A&F cycle facilitator.

**Practical implications:**

Research teams cannot be expected to be involved in implementing clinical care. Guidelines will keep being produced to improve service quality and will be expected to be practiced. This study gives insights into planning and tailoring A&F cycles.

**Originality/value:**

Tools to ease implementation are not enough, and the key seems to lie with facilitating a process using A&F. This study underscores leadership, designated responsibility and facilitation throughout a full audit cycle.

## Introduction

**Figure 1:**
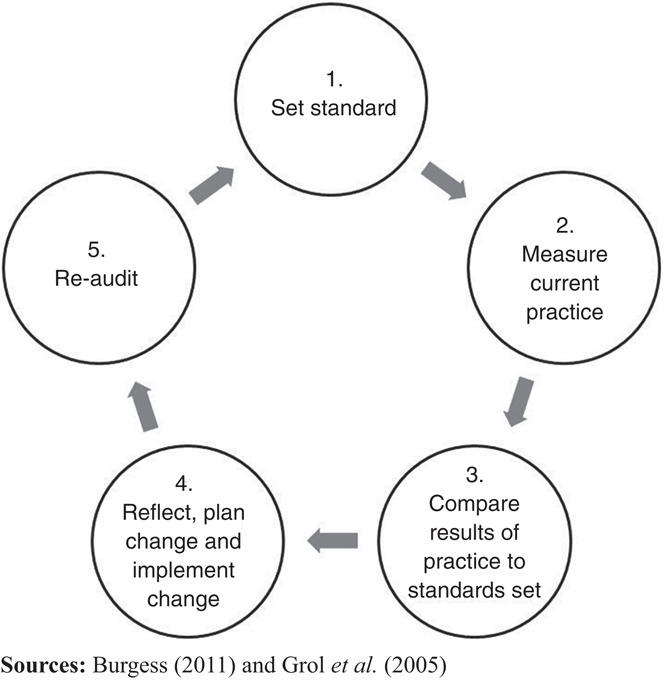
A common, modified audit and feedback (A&F) cycle

Evidence-based clinical practice guidelines aim to secure and improve care for clients, patients and service users, and are produced at a fast pace in mental health services (Shekelle *et al.*, 2012; Barbui *et al.*, 2014). They are, however, not easily translated into practice (Grol, 2008; Girlanda *et al.*, 2017) and a gap between evidence summarized in guidelines (what is known) and what is adopted in practice (what is done) exists (Sederer, 2009). In recent years, more research on how to close the gap has emerged (Grimshaw *et al.*, 2012), but we still lack knowledge about how to choose the most appropriate strategies for guideline implementation (Gagliardi and Alhabib, 2015). One research field is the various implementation strategies believed to affect either practitioner behavior or patient outcome. One intervention is audit and feedback (A&F), an implementation strategy targeting healthcare professionals (Grimshaw *et al.*, 2012; Mazza *et al.*, 2013; Effective Practice and Organisation of Care, 2015). A&F, a well-established intervention that aims to improve healthcare quality, used in different healthcare settings, may positively influence compliance with desired practice (Foy *et al.*, 2005; Burgess, 2011; Hysong *et al.*, 2006; Ivers *et al.*, 2012; Ivers, Grimshaw, Jamtvedt, Flottorp, O’brien, French, Young and Odgaard-Jensen, 2014). A&F are used for heterogeneous interventions and involve developing clinical performance summaries (audit) over a specific time, and subsequently providing a summary (feedback) to individual practitioners, teams, or healthcare organization managers (Brehaut and Eva, 2012). A&F are often the basis for quality improvement (QI) or implementation projects, and can be described as a circular process, an audit cycle or A&F cycle (Burgess, 2011; Potter *et al.*, 2010; Dixon *et al.*, 2011) (Figure 1).

A&F are regularly designed to be a multifaceted improvement strategy (Grol *et al.*, 2005) where reflecting on results, agreeing on where improvement is needed and producing an action plan are essential processes (Burgess, 2011). The idea is that when performance and care are measured against evidence-based standards, feeding back results can be expected to motivate service providers to improve (Hysong *et al.*, 2017). A Cochrane review (140 trials) showed highly variable A&F, ranging from substantial positive effects to null and even negative effects on provider behavior (Ivers *et al.*, 2012). Variability may partly be caused by misunderstanding the causal mechanisms underlying A&F (Brehaut and Eva, 2012) or feedback characteristics (Ivers, Grimshaw, Jamtvedt, Flottorp, O’brien, French, Young and Odgaard-Jensen, 2014). Other explanations and modifiers could be organizational culture and feedback actionability (Hysong *et al.*, 2006), or defined barriers like under resourcing, project design inexperience or lacking an overall audit plan (Johnston *et al.*, 2000).

Foy *et al.* (2005) claimed that A&F will continue to be an unreliable approach to QI until we learn how and when it works best. Over time, research has attempted to understand A&F (Ivers, Grimshaw, Jamtvedt, Flottorp, O’brien, French, Young and Odgaard-Jensen, 2014), which is important, given the strategy’s popularity, and effect studies are necessary to understand the A&F cycle. There is, however, also a need to understand how an A&F cycle is experienced and used by healthcare professionals to enhance A&F understanding in real-life settings. Qualitative studies have inquired into how A&F is perceived. The A&F response is usually better when it was perceived relevant and the process fitted with provider preferences, local policies and values (Christina *et al.*, 2016; Kristensen and Hounsgaard, 2014). In concordance with quantitative research, facilities with a successful guideline adherence tend to deliver more timely, individualized and non-punitive feedback (Hysong *et al.*, 2006). Some authors (Ivers, Barnsley, Upshur, Tu, Shah, Grimshaw and Zwarenstein, 2014; Payne and Hysong, 2016; Kristensen and Hounsgaard, 2014) addressed the process and to have a process facilitator, which are amongst recommendations designed to accomplish a better A&F cycle. Clinicians also feel that A&F is fragmented and variable in its effectiveness, and they might feel disconnected (Sinuff *et al.*, 2015). Payne and Hysong (2016) observe that the assessment process itself generates emotions within physicians, which has an impact on which actions they take. Competing goals either at the organization or patient level are a barrier (Ivers, Barnsley, Upshur, Tu, Shah, Grimshaw and Zwarenstein, 2014; Payne and Hysong, 2016; Meehan *et al.*, 2006). In mental health services, Meehan *et al.* (2006) reports on topics related to A&F as ambivalence, competing work demands, weak support from senior medical staff, questionable evidence to support outcome measures, and that at eight months post implementation, nurses remained ambivalent about the outcome measurement’s benefits, and had not engaged in the process. To our knowledge, there are few in-vivo studies on how a full A&F cycle in mental health services is undertaken and experienced. Implementation and QI projects are facilitated by researchers, and there is a need to explore these processes as naturally occurring in clinical settings undertaken by local groups (Dogherty *et al.*, 2014). Our aim was to describe and explore how specialist mental health services staff experience working with an A&F cycle as a basis for implementation to gain real-life process knowledge.

### Background

Studies show a high comorbidity between substance use and mental health disorders, well documented through clinical and epidemiological studies (Grant *et al.*, 2004; Saban and Flisher, 2010; Morisano *et al.*, 2014; Landheim *et al.*, 2003; Lai *et al.*, 2015). Norwegian health authorities have over the last 30 years recognized and acknowledged dual disorder patients and the insufficient treatment they have been offered. Several reports have been published, and at last a national clinical guideline exists. The Norwegian National Guideline for Assessment, Treatment and Social Rehabilitation of Persons with Concurrent Substance Use Disorders and Mental Disorders (hereafter the “Guideline”) was launched in 2012 (The Norwegian Directorate of Health, 2012). It contains 93 recommendations covering user involvement, families, assessment, treatment, follow-up care, roles and responsibilities, with a separate chapter on implementation. The Guideline was developed for healthcare providers in either primary or specialist services, which targeted people with severe and less severe mental illness concurrent with substance misuse when the two disorders are associated with significant impairment.

The Norwegian National Advisory Unit on Concurrent Substance Abuse and Mental Health Disorders (hereafter the “Advisory Unit”) developed tools to assist Guideline implementation based on the Grol *et al.* (2005) change model, briefly describing an implementation process in stages. The Guideline was published in paper and electronic versions linked to recommended screening tools. A user panel chose the ten most important recommendations to be published as a pamphlet, poster and electronic versions. Other implementation initiatives were developed; e.g., website (www.snakkomrus.no) and national learning program. The main strategy was an audit survey mirroring the most important Guideline recommendations. The survey (designed to be applicable to audit practice in District Psychiatric Centres (DPCs)), included 46 questions about screening and diagnostic practices, assessing the target group, integrated treatment, collaboration, evidence-based methods and competence requirements. It was an electronic self-report questionnaire, which came with a standardized action form to be completed and a template for its use. The action form recommended improvements, goals, actions, progress plan, main responsibility, economic assessment and evaluation. The audit survey with templates was available to DPCs on the Advisory Units website (Norwegian National Advisory Unit on Concurrent Substance Abuse and Mental Health, 2013).

## Method

### Study design

The study was a naturalistic, not a research-driven process, which aimed to learn from an A&F cycle decided and led by DPC unit leaders. The study had a descriptive and explorative design. A qualitative method with focus group interviews was chosen to understand A&F experiences.

### Study site

As a naturalistic study, we contacted DPC staff who might be about to initiate a QI process by using pre-existing A&F with action forms to implement the Guideline. Staff in one South-Eastern Norway DPC were ready to start in 2014 and agreed to participate. The DPC was an independent unit within a hospital trust, with responsibility for providing specialist mental health services to adults in one geographical area. The DPC included one general outpatient unit, one crisis resolution team and two inpatient units, offering services to a 72,000 population.

### The local audit process

A supervisor at the health trust with responsibility for information on services in concurrent substance abuse and mental health disorders held an introductory educational meeting at the DPC, spring, 2013. Later the same year, she was present at a management meeting at the hospital trust, on the Guideline, tools for implementation and audit survey. A decision was made to start the implementation process. Existing practice was audited in March 2014 with the supervisor’s assistance. All four units were included. Feedback was given at unit level verbally and in writing about four weeks after the audit. Each unit held separate meetings where all staff were invited to sessions on evidence-based practice, recommendations in the Guideline and on how to conduct an implementation process informed by an A&F cycle. The meetings were led by the supervisor with unit leaders present.

QI teams were formed, chosen by unit leaders, with one to three QI teams per unit. Seven teams with 6–21 members (53 participants) were included. The QI teams reflected unit staff; the two inpatient units included nurses, social educators or nursing assistants and one psychiatrist or psychologist. The QI crisis resolution team had similar education and roles, held more meetings and all staff were included. The general outpatient clinic team had more doctors, psychologists and specialist nurses, working regular hours with booked appointments. Only the crisis resolution team had the unit leader present at the QI team meetings.

The QI teams gathered three to four weeks after feedback to reflect on the results and to complete the action plans for the following year. A final decision on the improvement areas and the action plans was made in a DPC management meeting in June 2014, and the following areas were chosen: alcohol and drug use screening; concurrent substance abuse and mental illness treatment; and improving collaboration between DPC, substance abuse departments in the health authority and local authorities.

The actions (the implementation phase) were initiated the following year with unit leaders responsible for their completion. Amongst the actions were educational meetings aimed at all personnel, inter-professional meetings to enhance knowledge and possible role expansion. There were plans to enhance and improve communication and shared care between different service providers and making available educational materials. A re-audit led by the supervisor was conducted in June 2015; all units participated. Feedback was given to the DPC managers (DPC head and unit leaders) in June 2015 to be forwarded to the staff immediately afterwards by the unit leaders.

### Recruitment, setting and description of the focus groups

Focus group participants were recruited from QI team members appointed at the outset a year earlier. Members were selected by the unit leaders at our request from QI team participants representing several occupational groups. All participants were health professionals working with patients daily. Each group included four to six participants from the same unit, with the aim to homogenize experience and context within the group. Consequently, the groups included participants with different educational and professional backgrounds, such as psychologists, specialist nurses and social educators, which provided rich data (Kitzinger, 1995), totaling 20 persons. No unit leader was present in the focus groups, which allowed staff to speak more freely.

### Ethical approval and consent to participate

Participation in the audit and QI team creation were initiated and executed by the DPC. Information about the research project was given orally and in writing to all group members, and all participants signed a consent form. Since staff were asked by their leader to take part in the focus groups, we cannot be certain that participation was always consensual. The study was supported by the Innlandet Hospital Trust managers and approved by the Data Protection Officer for Research at Oslo University Hospital (Table I).

### Data collection

We conducted one focus group interview in each unit when the full A&F cycle was completed. An interview guide focusing on the complete A&F cycle was created, drawing on QI team meeting observations in 2014, and on A&F cycle literature (Potter *et al.*, 2010; Ivers, Sales, Colquhoun, Michie, Foy, Francis, and Grimshaw, 2014). The interview guide was created by the first author around A&F usefulness, action schema, actions taken to implement the National Guideline. We ensured the groups were asked similar questions to avoid inconsistency. Areas open enough to ensure that we got insight into the phenomenon (Graneheim and Lundman, 2004). Two focus groups interviews took place in June 2015 and two in September 2015. Interviews lasted approximately one hour and were led by the first author, helped by an experienced moderator whose role was to ensure that every group member was heard. The conversation focused on the study aim. Main points summarized to ensure validation. Summarizing was done by the moderator. Participants were given the opportunity to make changes or add additional comments. Follow-up questions ascertained whether information was understood. Interviews were audio recorded and summarized immediately after each interview, including reflections.

### Analysis

Our qualitative content analysis used several steps, following Graneheim and Lundman (2004) – a method suitable for analyzing communication systematically; it focuses on context and subject matter and emphasizes both similarities and differences. The aim is to condense and describe the phenomenon and to establish categories sharing the same meaning (Elo and Kyngäs, 2008). Since our purpose was to learn more about how an A&F cycle is experienced by mental health service staff, we decided on an inductive content analysis with no pre-existing analytical framework, which is recommended when there is insufficient or fragmented knowledge about the phenomenon (Elo and Kyngäs, 2008), but had the main areas from the interview guide as an outset for the analysis. The audio-recorded interviews were transcribed verbatim and NVivo 10 qualitative data analysis software was used to facilitate analysis. Materials were read several times to sense the whole, and to gather ideas for further analysis. Text was divided into meaning units, which were examined and condensed with the key content preserved and coded. The codes were sorted into categories grounded in the data, representing a manifest content in the text (Graneheim and Lundman, 2004). The categories were discussed and revised by the research team to enhance credibility.

## Findings

Themes evolved around three areas in the interview guide. The first was A&F usefulness and how staff experienced A&F, which was experienced as meaningful and reassuring. The second area was experience around the actions schema and the actions taken to implement Guideline recommendations; staff experienced poor follow-up on actions and poor prioritization. The last area was broader experience; staff felt they did not own the full A&F cycle.

### Audit was experienced as meaningful and reassuring

Staff valued being audited in two ways. Despite initial uncertainty, especially amongst outpatient unit and crisis resolution team participants, there was no resistance to participate in the survey. All participants found it meaningful to have their practice audited. Some pointed out that it probably had more value as a management tool to understand what training courses were needed, who needed to be supported to achieve goals and so on. When they talked about A&F in less direct ways, they often revealed that the value was obvious and helped to ensure service quality. Most groups said A&F was a way to increase awareness about practice and about patients with dual diagnosis, their rights, and the treatment methods recommended in the Guideline. One group also talked about increased awareness leading to a stronger responsibility sense. Patients with a dual diagnosis were commonly passed from one unit to another, often from mental health services to drug treatment services. The Guideline clarifies who is responsible for integrated treatment in mental health services:

Well, I think the fact that this [A&F] comes from outside the unit leads to awareness and so a change of attitude. The fact that these patients have been passed around in the systems is a proof of our individual responsibility.

One focus groups (an inpatient unit) found A&F particularly useful, especially in providing reassurance about current practice and reinforcing what they already knew. It was a way to boost informal or hidden competencies and make them visible and useful. This was viewed as adding value to the whole unit, and brought forth pride in the staff, and competencies from which everyone could benefit:

When I started here two years ago, I thought several of the staff here had great knowledge of substance abuse. They had never been acknowledged for the expertise they had and that it was good enough. In that way, this A&F was terrific. We got it in writing that there is competence! Yes, we have potential for improvement, but there was actually a lot that was of good quality and I think that was great!

All groups agreed that for an A&F to have any value, it must be followed by something more. It must be a process, followed by discussions on where to improve practice and action plans to achieve goals. In this process, QI teams discussed feedback and drew up action plans, and thus the A&F cycle was experienced as meaningful:

It’s supposed to be an audit, and then some actions and then a re-audit. There’s no help in just auditing and auditing and just waiting and seeing.

### Follow-up actions

Planned actions tended to fizzle out. Participants from three groups reported having started enthusiastically. They drew up action plans, but after a while, they felt that planned actions became just that: planned. As an example, one inpatient unit group described an action to become familiar by reading the Guideline. Lists were made to be signed after reading. After a few weeks, about half the staff had signed, but then nothing happened. Participants missed having a person with the responsibility to follow-up actions they planned and there was no one to ensure that everyone mutually understood the implementation:

Can they just tell us [that] we should read the National Guideline and then everybody does that? It’s not like that. I think the leader had a list in his office, and when you’d read the National Guideline you ticked off your name. It just came to nothing.

When asked about what they as staff members missed or would suggest as A&F cycle improvements, the groups primarily requested a reminder function, meaning someone with the function and the responsibility to execute the actions, to systematize what needed to be done, and to ensure that actions were implemented. No group member stated that unit leaders kept a firm hand on the process, or that DPC practice and development nurses had a significant role. In other words, staff experienced that no one facilitated the full A&F cycle. A halfway stage evaluation was a suggestion that could have made the implementation process better, which was considered a possible and necessary way to complete the A&F cycle, where the improvement areas chosen would have been implemented. Staff in three focus groups reflected on the time that passed between actions being set out and anyone asking for results or reminding them of the process. Their proposed solution was a much more forceful follow-up, or a supervisor or facilitator throughout the A&F cycle:

We need a reminder, someone to be in charge. That could be a practice and development nurse who can be in charge throughout the year, not just set up meetings.

When asked specifically about unit leader role, all groups experienced poor follow-up. Staff in two groups understood the leader’s time commitments and heavy workload, and sometimes seemed even to excuse the unit leader:

But the unit leader has tried in a way […] as far as she could in our everyday situation.

### Non-prioritization

Participants found that new focus areas constantly appeared, needing to be addressed and implemented. There was a flow of new national guidelines, local procedures and projects. It was considered overwhelming and fragmented and as continuously coming from “above.” They felt that they did not have the opportunity to learn anything well enough or incorporate it into practice. Since there was insufficient time for the changes to be practiced, recommendations easily disappeared; they were forgotten and not used in everyday treatment:

Yes, we’re talking about it, that now there’s yet another thing coming. We lose one and get another. We don’t have time to immerse ourselves. Something new comes along that we also have to deal with. And then it easily disappears along the way. So, we don’t forget the dual diagnosis patients, but we may not have the increased focus that we should have.

### A&F cycle ownership

Regarding the full A&F cycle process, there was no ownership among any focus group. They recalled certain courses, meetings and common interest in the beginning, but little sense of it as a process. They agreed that they had been invited to participate by filling out action plans and suggesting activities to achieve goals and admitted that they had subsequently been able to participate in occasional actions, but the implementation process or the A&F cycle as a whole was not owned. Some also questioned whether managers had a genuine interest and ownership in the process, stating that leaders needed to own the process and transfer it to staff. Outpatient clinic staff said that they did what they were asked to do, and that A&F was one among several things implemented without being part of something bigger. A&F was seen by some groups as imposed on them without the opportunity to discuss or reflect on its purpose, or how it best can form a basis for QI:

Well, I think if we, the ones doing the practical work, are to get ownership […] we should be involved. We should have a say.

Re-audit feedback is supposed to be given immediately after its completion, but when asked, it turned out that no staff, only managers, had received feedback from the re-audit, one to three months after completion.

## Discussion

We found that staff felt accepting and were mainly positive in the initial A&F cycle stages, were willing to participate in the audit survey, and implementation, but everyday life in the DPC units interfered with completion. Plans were often not fulfilled owing to organizational deficiencies and poor follow-up, and thus lacking A&F cycle ownership. Auditing raised practice awareness about dual diagnosis patients, in line with A&F intentions (Ivers, Sales, Colquhoun, Michie, Foy, Francis, and Grimshaw, 2014; Jamtvedt *et al.*, 2006) – an important recognition by mental health staff.

Participants felt the need for someone to facilitate the implementation process, a common recommendation for an A&F cycle (Burgess, 2011), shown to be important in earlier qualitative studies (Kristensen and Hounsgaard, 2014). Difficulties focusing on QI, when organizational resources are not established or available are a known barrier (Ivers, Barnsley, Upshur, Tu, Shah, Grimshaw and Zwarenstein, 2014). A systematic review examined the facilitator in the implementation process (Dogherty *et al.*, 2014), which showed that in most studies, there is an external researcher or research team responsible for implementation processes. They questioned how results are transferable to a clinical context without external researchers present. The study showed that it was probably hard to hold on to a full A&F cycle without designated facilitators.

Advisory Unit staff produced the audit survey and associated action schemas to ease Guideline implementation, but as it seems, it was not enough. We know from other studies that during the implementation phase, support from a local leader and at department level is crucial for guideline success in mental healthcare (Forsner *et al.*, 2010).

Unit leaders and department managers seemed to have failed to sell A&F as a useful improvement tool; its purpose and goal were not clear to staff, resulting in non-ownership. On the other hand, focus group participants were the same as those in the QI teams one year earlier, at the outset. We expected this to have given some ownership; one mental health service study, found multidisciplinary team participation to result in implementation phase ownership, and was seen to facilitate success (Forsner *et al.*, 2010). It seems that unit leaders were not able to follow-up all the way through a full A&F cycle, and staff felt overwhelmed by non-prioritization, and did not grab the chance given by QI teams to be an active part in the process.

When interviews were conducted, participants had been using the A&F cycle for one year to implement Guideline recommendations. Outpatient clinic staff had implemented several other important changes, like getting a new unit leader and moving to a new location that integrated with the DPC. A large reorganization in the health authority also led to additional work for the DPC along with restrictions on the educational budgets, which might have restrained the full A&F cycle.

We cannot expect research teams to be involved in clinical project implementation and must expect specialist mental health service staff to take autonomous responsibility for implementing national guidelines. Guidelines will, with great probability, keep being produced by national authorities to improve care quality, who expect them to be practiced. Tools to ease implementation are not enough. The key seems to lie with facilitating an implementation process.

### Strengths and limitations

The study’s strength is the inductive and naturalistic study design, which provided insight into how the A&F cycle is used in clinical practice to implement the Guideline, and how this is experienced by staff in Norway’s mental health services. Given the study’s naturalistic approach, a limitation is the difficulty replicating the study design, involving the entire process. We sought to strengthen validity by describing thoroughly the local A&F cycle and methods used. The material’s richness might be limited to one site, but we included all units to increase heterogeneity and richness. The interviews were not piloted since ours was a naturalistic study.

Interviews were conducted a year after the initial A&F, after actions were taken to implement Guideline recommendations and after a re-audit. This was to get the full A&F cycle experience. Nevertheless, recall bias is a possibility when reflecting on a process over an entire year. We accounted for this by going through the process at each interview.

Focus groups allow interactions, relationships and discussions among the participants (Kitzinger, 1995). There are, however, also limitations regarding stronger and weaker voices in the group when group members come from the same unit and are colleagues; i.e., professional hierarchies or group norms not accepted by everyone. We sought to compensate by having an experienced moderator who focused on this potential problem. The first author and interviewer is an information scientist with a speciality in health service organizations and evidence-based practice.

## Conclusion and recommendations

Exploring staff experiences provides valuable information regarding A&F cycles. This information may be used when planning and tailoring further implementation projects. We found a process that was formally decided at high management levels in the organization, taken on by local managers and given support in the initial phase. The A&F cycle was well planned and set out. Our main findings are that staff felt enthusiastic in the A&F cycle’s initial stages, but that everyday life in the DPC units interfered with the process and good intentions were often not fulfilled owing to organizational deficiencies and poor follow-up. This study shows that it is probably hard to hold on to a full A&F cycle without designated facilitators even when tools for implementation are ready at hand. For healthcare organizations, this gives valuable insight into staff needs and facilitation. Our study is relevant for leaders and managers in specialist mental health services using A&F as tool to implement guidelines and promote evidence-based practice. We suggest exploring how unit leaders in mental health services experience the process and complete an A&F cycle. We recommend more naturalistic studies in specialist mental health services to understand A&F processes in real life.

## Figures and Tables

**Table I:** Quality improvement teams (QI teams) and focus groups

	QI teams (members)^a^	Focus group members (number participated in a QI team meeting 1 year earlier)^b^	Educational background: focus groups members^c^
General outpatient clinic	2 (14)	6 (5)	Psychologist specialists, psychologists, special nurses
Inpatient unit 1	3 (21)	5 (5)	Psychologist, nurses, assistant nurse, social worker
Inpatient unit 2	1 (6)	4 (3)	Psychologist, special nurse, social educator, assistant nurse
Crisis resolution team	7 (12)	5 (4)	Nurses and special nurses

**Notes:** ^a^QI teams and total members from each unit; ^b^focus groups members and how many participated in the QI teams one year later; ^c^educational background

## References

[ref001] Barbui C., Girlanda F., Ay E., Cipriani A., Becker T. and Koesters M. (2014), “Implementation of treatment guidelines for specialist mental health care”, Cochrane Database of Systematic Reviews, Vol. 1 No. CD009780, pp. 1-43. 10.1002/14651858.CD009780.pub2 24443146

[ref002] Brehaut J.C. and Eva K.W. (2012), “Building theories of knowledge translation interventions: use the entire menu of constructs”, Implementation Science, Vol. 7 No. 114, pp. 1-10, doi: 10.1186/1748-5908-7-114. PMC3520870 23173596

[ref003] Burgess R. (2011), New Principles of Best Practice in Clinical Audit, Radcliffe Publishing, Oxford.

[ref004] Christina V., Baldwin K., Biron A., Emed J. and Lepage K. (2016), “Factors influencing the effectiveness of audit and feedback: nurses’ perceptions”, Journal of Nursing Management, Vol. 24 No. 8, pp. 1080-1087. 27306646 10.1111/jonm.12409

[ref005] Dixon N., Pearce M. and Quest H.Q. (2011), Guide to Using Quality Improvement Tools to Drive Clinical Audits, HQIP, London, available at: www.hqip.org.uk/resources/hqip-guide-to-using-quality-improvement-tools-to-drive-clinical-audit/ (accessed July 2017).

[ref006] Dogherty E.J., Harrison M., Graham I. and Keeping-Burke L. (2014), “Examining the use of facilitation within guideline dissemination and implementation studies in nursing”, International Journal of Evidence-Based Healthcare, Vol. 12 No. 2, pp. 105-127. 24945960 10.1097/XEB.0000000000000008

[ref007] Effective Practice and Organisation of Care (2015), “EPOC taxonomy”, available at: https://epoc.cochrane.org/epoc-taxonomy (accessed July 6, 2017).

[ref008] Elo S. and Kyngäs H. (2008), “The qualitative content analysis process”, Journal of Advanced Nursing, Vol. 62 No. 1, pp. 107-115. 18352969 10.1111/j.1365-2648.2007.04569.x

[ref009] Forsner T., Hansson J., Brommels M., Wistedt A.A. and Forsell Y. (2010), “Implementing clinical guidelines in psychiatry: a qualitative study of perceived facilitators and barriers”, BMC Psychiatry, Vol. 10 No. 8, pp. 1-10, doi: 10.1186/1471-244X-10-8. 20089141 PMC2822755

[ref010] Foy R., Eccles M.P., Jamtvedt G., Young J., Grimshaw J.M. and Baker R. (2005), “What do we know about how to do audit and feedback? Pitfalls in applying evidence from a systematic review”, BMC Health Services Research, Vol. 5 No. 50, pp. 1-7, doi: 10.1186/1472-6963-5-50. 16011811 PMC1183206

[ref011] Gagliardi A.R. and Alhabib S. (2015), “Trends in guideline implementation: a scoping systematic review”, Implementation Science, Vol. 10 No. 54, pp. 1-11, doi: 10.1186/s13012-015-0247-8. 25895908 PMC4409784

[ref012] Girlanda F., Fiedler I., Becker T., Barbui C. and Koesters M. (2017), “The evidence-practice gap in specialist mental healthcare: systematic review and meta-analysis of guideline implementation studies”, The British Journal of Psychiatry, Vol. 210 No. 1, pp. 24-30. 27445353 10.1192/bjp.bp.115.179093

[ref013] Graneheim U.H. and Lundman B. (2004), “Qualitative content analysis in nursing research: concepts, procedures and measures to achieve trustworthiness”, Nurse Education Today, Vol. 24 No. 2, pp. 105-112. 14769454 10.1016/j.nedt.2003.10.001

[ref014] Grant B.F., Stinson F.S., Dawson D.A., Chou S.P., Dufour M.C., Compton W., Pickering R.P. and Kaplan K. (2004), “Prevalence and co-occurrence of substance use disorders and independent mood and anxiety disorders: results from the national epidemiologic survey on alcohol and related conditions”, Archives of General Psychiatry, Vol. 61 No. 8, pp. 807-816. 15289279 10.1001/archpsyc.61.8.807

[ref015] Grimshaw J.M., Eccles M.P., Lavis J.N., Hill S.J. and Squires J.E. (2012), “Knowledge translation of research findings”, Implementation Science, Vol. 7 No. 50, pp. 1-17, doi: 10.1186/1748-5908-7-50. PMC3462671 22651257

[ref016] Grol R. (2008), “Knowledge transfer in mental health care: how do we bring evidence into day-to-day practice?”, Canadian Journal of Psychiatry, Vol. 53 No. 5, pp. 275-276. 18551848 10.1177/070674370805300501

[ref017] Grol R., Wensing M. and Eccles M. (2005), Improving Patient Care: The Implementation of Change in Clinical Practice, 1st ed., Elsevier, Edinburgh.

[ref018] Hysong S.J., Best R.G. and Pugh J.A. (2006), “Audit and feedback and clinical practice guideline adherence: making feedback actionable”, Implementation Science, Vol. 1 No. 9, pp. 1-10, doi: 10.1186/1748-5908-1-9. PMC1479835 16722539

[ref019] Hysong S.J., Kell H.J., Petersen L.A., Campbell B.A. and Trautner B.W. (2017), “Theory-based and evidence-based design of audit and feedback programmes: examples from two clinical intervention studies”, BMJ Quality & Safety, Vol. 26 No. 4, pp. 323-334. 10.1136/bmjqs-2015-004796 27288054

[ref020] Ivers N., Barnsley J., Upshur R., Tu K., Shah B., Grimshaw J. and Zwarenstein M. (2014), “My approach to this job is … one person at a time’: perceived discordance between population-level quality targets and patient-centred care”, Canadian Family Physician, Vol. 60 No. 3, pp. 258-266. 24627384 PMC3952764

[ref023] Ivers N.M., Sales A., Colquhoun H., Michie S., Foy R., Francis J.J. and Grimshaw J.M. (2014), “No more ‘business as usual’ with audit and feedback interventions: towards an agenda for a reinvigorated intervention”, Implementation Science, Vol. 9 No. 14, pp. 1-8, doi: 10.1186/1748-5908-9-14. 24438584 PMC3896824

[ref021] Ivers N.M., Grimshaw J.M., Jamtvedt G., Flottorp S., O’brien M.A., French S.D., Young J. and Odgaard-Jensen J. (2014), “Growing literature, stagnant science? Systematic review, meta-regression and cumulative analysis of audit and feedback interventions in health care”, Journal of General Internal Medicine, Vol. 29 No. 11, pp. 1534-1541. 24965281 10.1007/s11606-014-2913-y PMC4238192

[ref022] Ivers N.M., Jamtvedt G., Flottorp S., Young J.M., Odgaard-Jensen J., French S.D., O’brien M.A., Johansen M., Grimshaw J. and Oxman A.D. (2012), “Audit and feedback: effects on professional practice and healthcare outcomes”, Cochrane Database of Systematic Reviews, Vol. 6, p. 229. 10.1002/14651858.CD000259.pub3 PMC11338587 22696318

[ref024] Jamtvedt G., Young J.M., Kristoffersen D.T., O’brien M.A. and Oxman A.D. (2006), “Does telling people what they have been doing change what they do? A systematic review of the effects of audit and feedback”, Quality & Safety in Health Care, Vol. 15 No. 6, pp. 433-436. 17142594 10.1136/qshc.2006.018549 PMC2464905

[ref025] Johnston G., Crombie I.K., Davies H.T., Alder E.M. and Millard A. (2000), “Reviewing audit: barriers and facilitating factors for effective clinical audit”, Quality in Health Care, Vol. 9 No. 1, pp. 23-36. 10848367 10.1136/qhc.9.1.23 PMC1743496

[ref026] Kitzinger J. (1995), “Qualitative research: introducing focus groups”, British Medical Journal, Vol. 311 No. 7000, pp. 299-302. 7633241 10.1136/bmj.311.7000.299 PMC2550365

[ref027] Kristensen H. and Hounsgaard L. (2014), “Evaluating the impact of audits and feedback as methods for implementation of evidence in stroke rehabilitation”, British Journal of Occupational Therapy, Vol. 77 No. 5, pp. 251-259.

[ref028] Lai H.M., Cleary M., Sitharthan T. and Hunt G.E. (2015), “Prevalence of comorbid substance use, anxiety and mood disorders in epidemiological surveys, 1990-2014: a systematic review and meta-analysis”, Drug and Alcohol Dependence, Vol. 154, September, pp. 1-13. 26072219 10.1016/j.drugalcdep.2015.05.031

[ref029] Landheim A.S., Bakken K. and Vaglum P. (2003), “Gender differences in the prevalence of symptom disorders and personality disorders among poly-substance abusers and pure alcoholics. Substance abusers treated in two counties in Norway”, European Addiction Research, Vol. 9 No. 1, pp. 8-17. 12566793 10.1159/000067732

[ref030] Mazza D., Bairstow P., Buchan H., Chakraborty S.P., Van Hecke O., Grech C. and Kunnamo I. (2013), “Refining a taxonomy for guideline implementation: results of an exercise in abstract classification”, Implementation Science, Vol. 8 No. 32, pp. 1-10, doi: 10.1186/1748-5908-8-32. 23497520 PMC3606141

[ref031] Meehan T., Mccombes S., Hatzipetrou L. and Catchpoole R. (2006), “Introduction of routine outcome measures: staff reactions and issues for consideration”, Journal of Psychiatric and Mental Health Nursing, Vol. 13 No. 5, pp. 581-587. 16965478 10.1111/j.1365-2850.2006.00985.x

[ref032] Morisano D., Babor T. and Robaina K. (2014), “Co-occurrence of substance use disorders with other psychiatric disorders: implications for treatment services”, Nordic Studies on Alcohol and Drugs, Vol. 31 No. 1, pp. 5-25.

[ref033] Norwegian National Advisory Unit on Concurrent Substance Abuse and Mental Health (2013), “Gap-undersøkelse for behandlere i psyksik helsevern (audit-survey for health professionals in specialist mental health services)”, Norwegian National Advisory Unit on Concurrent Substance Abuse and Mental Health, Ottestad, available at: http://gap.rop.no/skjemamaler/for-behandlere-i-psykisk-helsevern.-versjon-2.0 (accessed July 6, 2017).

[ref034] Payne V.L. and Hysong S.J. (2016), “Model depicting aspects of audit and feedback that impact physicians’ acceptance of clinical performance feedback”, BMC Health Services Research, Vol. 16 No. 260, pp. 1-12, doi: 10.1186/s12913-016-1486-3. 27412170 PMC4944319

[ref035] Potter J., Fuller C. and Ferris M. (2010), Local Clinical Audit: Handbook for PHYSICIANS, HQIP, London, available at: www.hqip.org.uk/assets/Guidance/Local-clinical-audit-handbook-for-physicians-August-2010-FINAL.pdf (accessed October 2, 2015).

[ref036] Saban A. and Flisher A.J. (2010), “The association between psychopathology and substance use in young people: a review of the literature”, Journal of Psychoactive Drugs, Vol. 42 No. 1, pp. 37-47. 20464805 10.1080/02791072.2010.10399784

[ref037] Sederer L.I. (2009), “Science to practice: making what we know what we actually do”, Schizophrenia Bulletin, Vol. 35 No. 4, pp. 714-718. 19460879 10.1093/schbul/sbp040 PMC2696375

[ref038] Shekelle P., Woolf S., Grimshaw Jeremy M., Schünemann Holger J. and Eccles Martin P. (2012), “Developing clinical practice guidelines: reviewing, reporting, and publishing guidelines; updating guidelines; and the emerging issues of enhancing guideline implementability and accounting for comorbid conditions in guideline development”, Implementation Science, Vol. 7 No. 62, pp. 1-7, doi: 10.1186/1748-5908-7-62. PMC3503794 22762242

[ref039] Sinuff T., Muscedere J., Rozmovits L., Dale C.M. and Scales D.C. (2015), “A qualitative study of the variable effects of audit and feedback in the ICU”, BMJ Quality & Safety, Vol. 24 No. 6, pp. 393-399. 10.1136/bmjqs-2015-003978 25918432

[ref040] The Norwegian Directorate of Health (2012), “National guideline for assessment, treatment and social rehabilitation of persons with concurrent substance use disorders and mental disorders”, The Norwegian Directorate of Health, Oslo, Norwegian National Guidelines, IS-1948, available at: https://helsedirektoratet.no/Lists/Publikasjoner/Attachments/188/Nasjonal-faglig-retningslinje-personer-med-rop-lidelser-IS-1948.pdf (accessed July 6, 2017).

